# The Effect of Cognitive–Behavioral Play Therapy on Improvements in Expressive Linguistic Disorders of Bilingual Children

**DOI:** 10.3389/fpsyg.2021.626422

**Published:** 2022-01-06

**Authors:** Shahrzad Rezaeerezvan, Hossein Kareshki, Majid Pakdaman

**Affiliations:** ^1^Department of Psychology, Qaenat Branch, Islamic Azad University, Qaenat, Iran; ^2^Department of Psychology, Faculty of Psychology & Educational Sciences, Ferdowsi University of Mashhad, Mashhad, Iran; ^3^Department of Psychology, Qaenat Branch, Islamic Azad University, Qaenat, Iran

**Keywords:** Cognitive-Behavioral Therapy, play, treatment, expressive language disorders, bilingual, children

## Abstract

The present study attempted to investigate the effect of cognitive-behavioral play therapy (CBPT) on the improvements in the expressive linguistic disorders of bilingual children. The population consists of all bilingual children with expressive linguistic disorders studying in preschools. Considering the study’s objectives, a sample of 60 people, in three groups (experimental, control, and pseudo-control), were selected using WISC, TOLD, and clinical interviews. The experimental group members participated in CBPT training sessions. The training consisted of twelve 90-min sessions, three times per week programs held every other day. The pseudo-control group received training different from play therapy. The experimental group members were subjected to the follow-up test 2 months after the end of the intervention. All three groups sat the TOLD3 test before and after the experiment. Data analysis was carried out using ANCOVA. The results of data analysis suggested that CBPT can improve the expressive language disorders of bilingual children.

## Introduction

There is no identical opinion in the definition of bilingualism between different researchers، Some researchers define bilingualism as complex psychological, linguistic, and sociocultural behavior that has different dimensions (Blom et al., 2014), whereas some researchers define bilingualism as the ability of bilinguals to use two different languages, especially in the verbal dimension (Martin et al., 2016). While the most comprehensive definition, accepted by almost all experts, is that bilingualism is a state in which a person is taught in a language other than his or her original language (Friso-Van Den Bos et al., 2013). Bilinguals face more problems than monolinguals in different levels of education, such as reading, writing, speaking, and even arithmetic, because their primary language differs from the official language taught in preschool and school (Barac and Bialystok, 2011). Bilingual persons generally experience several problems like linguistic, cognitive, and social development. Relevant literature has shown that an obvious mechanism that could account for bilingual disadvantages in tasks that focus on lexical processing is interference among languages (Gollan et al., 2005). On the other hand, Iran is a country consisting of different ethnic minorities speaking different languages. In this country, bilingualism is realized through formal education; that’s why some children master two languages very well, while others know one language better than the other (Aliabadi et al., 2018). Bilingual children not only have speech problems but also have fewer words than monolingual children, and their pronunciation is impaired (Isanejad and Alidadi, 2018). Calvo et al. propose that bilinguals have more difficulty in cognitive development than monolinguals (Calvo and Bialystok, 2014). The findings of a study conducted by Benner (2009) also suggest that 91.3% of students with any learning disorder (LD) also had a language disorder. Students with an emotional disorder who suffered from a reading disability also showed signs of language impairment (Mattison, 2008). Engel de Abreu et al. emphasized the greater learning difficulties of bilingual students than monolingual students and showed that if cognitive development and cognitive control in the group of bilingual students improves, it will improve their academic performance (Engel de Abreu et al., 2012). Thus, language is one of the most important elements of developmental learning that should develop in a child before primary school and is the basis for his social, economic, and educational life, and language disorders are one of the most controversial groups in the diagnostic category of communication disorders (Gillon et al., 2020). The main diagnostic characteristics of these disorders include language acquisition and use difficulties due to reduced vocabulary, limited sentence structure, and impairment in discourse. Language acquisition and use are dependent on both receptive and expressive skills. Linguistic disorders involve receptive and expressive language disorders; in other words, when a person has difficulty comprehending others, he is suffering from a receptive disorder, and when he has difficulty expressing his thoughts, desires, and feelings, he suffers from an expressive disorder. Sadock (2007) has also reported that the prevalence of expressive language disorder is high among families with learning disabilities; therefore, any language disorder or delay in this category may lead to the development of learning disabilities (Tafti and Abdolrahmani, 2014). Language and speech development is a critical stage of development in humans. The present study focuses on expressive language disorder. Relevant literature shows that students’ interaction with teachers and parents, their desire to play with peers and talk to them, are important factors that can play a role in their language development (Mendelsohn, 2002; Akhavan and Mousavi, 2007). The effect of educational programs and various activities on the development of expressive language skills in children has been studied by several researchers (Hoshina et al., 2017; Morgan et al., 2019).

Play therapy can help children express their negative thoughts and feelings through games (Han et al., 2017). One of the best play therapy approaches designed for preschoolers is play therapy with a cognitive–behavioral approach. This approach emphasizes the child’s participation in treatment. On the other hand, the cognitive-behavioral play therapy (CBPT) approaches to deal with behavior and thought changes; therefore, the objective of CBPT is to incorporate cognitive and behavioral techniques within a play therapy paradigm, which has emerged as the most effective treatment for children and adolescents with a range of disorders (Han et al., 2017). Previous studies have shown that preschool children with language disorders use less complex behaviors than normal children in terms of play quality, cooperation with peers, and the use of language in symbolic, adaptive, and mixed plays. Lewis et al., 2000; Gorovoy, 2008 also stated that certain aspects of play in young children are related to their emerging linguistic skills, and symbolic play has a significant correlation with the development of expressive language. Evidence suggests that cognitive–behavioral interventions can play an effective role in the treatment of speech and language disorders such as stuttering (Craig et al., 2002) and that CBPT can lead to the treatment of selective mutism (Knell, 1999). Moreover, Rabian and McCloskey (2010) investigated the effect of short-term cognitive–behavioral group therapy (CBGT) on the treatment of students with learning disabilities. They showed that CBGT could mitigate learning disabilities and improve students’ academic performance. Landreth et al. reported that play therapy could effectively improve children’s cognitive impairments with learning disabilities (Landreth et al., 2009). CBPT reduces learning disabilities in children with learning disabilities in reading (Malek et al., 2013). Moreover, play, music, and puppet shows can improve language learning and communication skills in preschool children (Ervin and Miller, 2012). A 19-year study (1993–2006) conducted on 427 German preschool children with language disorders showed that playing could significantly contribute to the treatment and diagnosis of children with language disorders (Ervin and Miller, 2012). Similarly, numerous studies have shown that play therapy interventions can improve speech and language disorders (Barlow, 1986; Brooks and Benjamin, 1989; Donahue-Kilburg, 1992; Danger and Landreth, 2005; Li, 2012; Mohammad Esmaeilzadeh et al., 2015) and play therapy improves the speech skills of bilingual children with language disorders (Riojas-Cortez and Flores, 2004).

A review of the background of research shows that the effectiveness of CBPT on the improvement of speech language disorders indicates that less research has been done on this issue, so the results of this study can pave the way for further research in this field. Inferring from the above explanations in the present study, a new and creative play therapy educational package whose goals, content, and tasks are completed in line with the cognitive and behavioral approach (storytelling, painting, presenting, and performing various cognitive and behavioral games, puppet shows, modeling and role-playing, and child reinforcement) was used. Since preschool is a critical period for language acquisition, developing programs tailored to their spirits and detecting effective communication techniques can be very fruitful. Therefore, the present study is with the aim of the effectiveness of cognitive and behavioral play therapy on improving the expressive language disorders of preschool bilingual children.

## Materials and Methods

To investigate the effect of an independent variable (play therapy) on the dependent variable (expressive language disorder), we design, pretest, posttest, and follow up steps and collect data from our studied control and pseudo-control groups.

### Population, Sample, and Sampling Method

The study population consisted of all bilingual preschool children with expressive language disorder who lived in Bojnourd city in 2019–2020. The number of students with expressive language disorder was specified through correspondence between the Department of Education and all the preschools of the city (110 students). Considering the study’s objectives, a sample of 60 students was selected using the Wechsler Intelligence Scale for Children [Wechsler Intelligence Scale for Children-Fourth Edition (WISC-IV; Wechsler, 2012)], and TOLD language test (test of language development. Primary, c1997, 3rd), clinical interview, and simple random sampling. The selected sample consisted of 30 girls and 30 boys, all of whom were 6 years old. The mean IQ of the experimental group was 87, the control group 85, and the pseudo-control group 88. Seventy percentage of the families have a low economic and social status, and 30% of them had a moderate level and none of the children had a good and very good social and economic status. Forty percentage of the parents were illiterate, 45% had primary education, and 15% had completed high school, while none of the parents had an academic or university education. The mother tongue spoken by the children included 35% Kurdish, 25% Turkish, and 40% Turkmen. Children’s primary language skills in vocabulary and word production were good and intermediate, but they were weak in grammatical comprehension; children’s L2 skills in both vocabulary and grammar were weak. The language assessment was based on the L2 because the official language of Iran is Persian, and although there may be several languages or dialects in a city, everyone should study in Persian after going to school. Therefore, the basis of language evaluation in this intervention is Persian language evaluation.

The children were assigned to three 20-member groups (experimental group, control group, and pseudo-control group). The experimental group members participated in CBPT training sessions (through a researcher-made intervention package whose psychometric properties had already been evaluated). The training consisted of 12 sessions, and each session lasted 90 min, three times per week programs held every other day. The pseudo-control group received training different from play therapy, and the control group did not receive any intervention during this period. The experimental group members were subjected to the follow-up test 2 months after the end of the intervention.

### Inclusion Criteria (Experimental Group)

Inclusion criteria were as follows: 1—being at least 6 years old; 2—intelligence score higher than 75 by Wechsler Intelligence Scale for Children; 3—the reception of no psychological and speech therapy services during participation in study; 4—lack of mental disorders (including hyperactivity and concentration control problems) and physical problems; 5—the standard language score is lower than 110 on the TOLD3 language test. Exclusion criteria were: 1—absence for more than two sessions; 2—physical and mental illness; 3—lack of adequate involvement in assignments and a lack of interest in play; 4—receiving any intervention or training during CBPT.

The instruments used to measure the dependent variable (improvement in expressive language disorder) were as follows:

### Data Collection Instrument

Wechsler Intelligence Scale for Children and TOLD language tests were used for data collection. In the present study, Test of Language Development (TOLD-P: 3), developed by Newcomer and Hamill (1997), was used to collect data about children’s expressive language performance (Newcomer and Hammil, 1997). This test has been adapted and standardized for Persian by Hassanzade and Minayi (2002). The average Cronbach’s alpha coefficients for different aspects of this test (listening, organizing, speaking, semantics, syntax, expressive language) fell within the 0.82–0.96 range (Faramarzi et al., 2015). This test is developed based on a two-dimensional model with one dimension dealing with linguistic systems (listening, organizing, and speaking) and the other dealing with linguistic coordinates (semantics, syntax, and phonology). This test contains nine subtests that can be administered to 4-0_ 8–11-year-old children. This test has been normed by Hasanzadeh and Minaie (Hassanzade and Minayi, 2002) that work on 1,235 children (609 girls and 626 boys). In this study, the receptive language, which covers the linguistic system of listening, was measured using a subtest of picture vocabulary and morphological comprehension. This test’s reliability was obtained using the internal consistency method (average alpha coefficient = 0.89). As for reliability, the correlation coefficients of the subtests calculated using the test–retest method were 0.78 and 0.82, respectively. The validity of this test was measured using content validity, criterion validity, and construct validity. As for the test’s validity, correlation coefficients between the subtests of the test and criterion tests (0.57, 0.71, 0.42, and 0.70) can be regarded as a measure of test validity (Vahab et al., 2012).

Wechsler Intelligence Scale for Children (WISC-V) was used to measure the intelligence of children. This scale has been adapted and normalized by Abedi et al. (2009) and Vahab et al. (2012). The subtest’s reliability ranged between 0.65 and 0.95 in the retest method and between 0.71 and 0.86 in the bisection method. This instrument was used as a screening tool to examine the inclusion criteria. Methodology: considering the student’s limited grasp of cognitive materials, attempts were made to make sure that the contents of group play therapy sessions are presented in simple language and tailored to students’ comprehension power.

The children’s expressive language skills were evaluated at the end of the intervention sessions and 2 months later to evaluate the stability of the effect of CBPT (Table 1).

**Table 1:** Designing CBPT sessions for bilingual children with expressive language disorders.

Session	The main purpose	Instrument	Activities	Implemented method
1	Interaction and familiarity of the child with the intervention method	Storytelling Self-portrait	Elaboration on the rules and activities to be covered during each session, effective communication, playroom and other students, introduction of scoring and bonus tables as well as homework assignments	Running the play “My Story”: with the aim of creating and expanding the relationship Drawing a picture of yourself: Aiming to better understand the child’s self-image. The therapist uses inductive questioning to guide the child to identify and distinguish thoughts, emotions, and activities
2	Relationships	Spoon dolls Grammar games	The enhancement of interpersonal and group communication skills, enhancement of concentration and listening through active participation, Socratic questioning, elaboration on the importance of emotions, behavioral techniques, introducing self-help exercises, and the important role and position of individuals in communications	Spoon Dolls: The purpose of this play is to examine the child’s relationship with others and the role of family members in the child’s life (children talk to each other in groups, the purpose of this is to increase the accuracy and attention of talking and listening) - Therapist using behavioral approach corrects and shapes children’s speech Grammar games: The child must do what he is told. These things are said in the form of poetry, such as: run-run or turn around and bring the book Self-help exercises is about two situations that the child identifies and discusses. Conversation focuses on the child’s internal reactions, including emotions, thoughts, and physical symptoms.
3	Empowerment and increase communication skills	What do you remember game Heterogeneous association table	The enhancement of interpersonal and group communication skills, using the cognitive–behavioral approach to empower picture vocabulary skills, employment of cognitive and behavioral techniques, project, role-playing, enhancement of verbal and nonverbal skills.	What do you remember game: we show the child a photograph of a trip, birth, visit to the zoo, etc., which is related to him, and we help him to tell the story to other children by using inductive method. Objective: To strengthen verbal and nonverbal skills, to create a deeper level of trust and understanding between the therapist and children, to teach deep breathing and to stop the contraction of the body when it becomes anxious when speaking. Heterogeneous association table: Children draw or color each of the table that have different and heterogeneous things and then make a story about it.
4	Self-recognition	Playing with Sand Playing the (Speaker Hat) Creation of stories	Introduction of self-recognition and control techniques, helping children differentiate between words through cognitive–behavioral approach, enhancement of assertiveness, enhancement of verbal and communicative skills, the introduction of self-esteem technique, development of self-monitoring skills, and real-time employment of a four-step confrontation plan	Playing with Sand: The child makes a picture with sand and then tells its story. Playing the (Speaker Hat): Children take turns putting their hats on their heads and answering questions. The activity of this session is recorded in the children’s workbook in an appropriate way. For example, a child’s work is photographed with a tray of sand and gravel, their date is recorded, and a title is assigned to each.
5	Self-awareness	Artwork (collage) Story recitation Puppet show	Emotional processing and consciousness, increasing the enhancement of syntactic aspects of linguistic communications using cognitive–behavioral approaches, self-help practice, practicing gradual exposure activities using elective methods, facilitation of real-time continuous self-monitoring, examination and enhancement of abilities and strengths, cognitive restructuring, enhancement and processing of self-knowledge skills.	Children make a drawing and tell a story about it using crayons, play dough, collage accessories, colored paper, and so on. Puppet show: The therapist starts an incomplete story with the doll in his hand and asks the children to finish the story using the dolls they have. End of activity: The therapist gives children the opportunity to complete the activity as they wish. For example, some children spray paint or line up their drawings. (Purpose: to feel in control of the situation)
6 & 7	Emotions	Playing Emotion Cycle Emotional Pantomime Storytelling Glossary of Emotions Facial impression plays	Emotion identification and management, empowering word imitation and auditory data processing skills using the cognitive–behavioral approach, enhancement of feeling expression skills and the tendencies to express emotional experiences, offering an effective confrontational model, practicing new confrontational skills, recognizing and expressing emotions in a safe environment, enhancement of concept comprehension and processing skills	Playing Emotion Cycle: A large circle that is divided into several parts and on each part of the image one of the types of emotions is displayed, then the child is asked to walk on the circle, and whenever we command the child to stop, the emotion that Imitate and explain standing on it Emotional Pantomime: This game is called “Emotion Guessing Game” or “Show without dialogue” to children. In this game, children have to guess the person’s feelings and the meaning of the person’s actions only according to the person’s facial movements and body postures. Storytelling: Objective: To identify and normalize emotions as coping, having different emotions in those situations and how to deal with them. This game includes self-talk that has been experienced in normalizing emotions. Glossary of Emotions: Children can create a dictionary of emotions using images of different emotions and paste these images in their book and name each one (happy apple, sad chair, angry child, etc.).
8	Physical reactions	Relaxing music Storytelling Modeling and role-playing Puppet Show	The identification of physical reactions and dealing with negative emotions, enhancing children’s ability to comprehend and express auditory concepts using a cognitive–behavioral approach, development of a model for dealing with wrong and non-adaptive reactions and beliefs about language disorders, identification of physical reactions, employment of Relaxation, self-regulation and self-talk techniques	Identification of physical reactions: Objective: To help children identify physical reactions related to emotions and situations. Perform the activity by telling a story about a bilingual child and the emotions he or she may encounter. Then the type of physical reactions is examined (performing the activity using questioning) An imaginary child is discussed and children express their ideas and opinions. And it tries to create hope in children to get the desired result. Modeling and role-playing: Children’s play. The therapist describes his or her emotional and physical responses and asks the children to describe their reactions.
9 & 10	Thoughts	Puppet Show Role-playing Making up stories with picture cards	Recognition of the role of thoughts, replacing negative and inefficient self-talk with positive self-talk, enabling children to enhance their oral vocabulary skills using a cognitive–behavioral approach, detection of non-adaptive beliefs during speaking and self-assertion, teaching oral vocabulary skills, differentiating between useful adaptive thoughts and non-adaptive ones	Storytelling: Purpose: Recognizing thoughts and identifying inner thoughts and self-talk Show Puppet: Purpose: differentiating between useful adaptive thoughts and non-adaptive ones Cartoon animation: Purpose: modeling coping skills, evaluating the correctness of thoughts. Roleplay: Performing a play using useful and confrontational inner self-talk
11	Problem-solving and decision making	Story making Playing Train	Problem-solving skills, increasing grammatical sentence completion skills using cognitive–behavioral approaches, enhancement of the ability to actively cope with negative thoughts and feelings, providing a confrontation model, active confrontational methods based on emotions and self-talk (Socratic method), problem-solving (Brainstorming method)	Performance of the play: Objective: Active confrontation with emotions and self-talk, Socratic questioning to challenge children’s beliefs (it is better for children with expressive language disorder to be spectators and children with perceived language disorder to play a role). Problem solving skills training: The therapist gives an example of daily tasks. Use the method (brainstorming) to gather ideas and encourage children to think about different ideas and choose the best option Play train: Children stand behind each other in the form of train carriages and stand at each station where the train whistle sounds and carry out the order. These commands include saying different words, completing a short sentence, expressing feelings about an event, and so on.
12	Evaluation and rewards	Puppet Show Facial expression cards	Development of self-initiated speaking using cognitive–behavioral approaches, introducing the concepts of reward and punishment, self-assessment and awarding, normalization of challenges and self-initiated speaking, giving a certificate of appreciation for the completion of treatment	Problem-solving skills and completing the intervention process Puppet show: Purpose: The concept of reward and punishment is explained. Increase the power of empathy Storytelling with picture cards: Purpose: self-assessment and self-reward Performance of “My Life Story”: Objective: To normalize challenges, spontaneously talk to children, positive self-assessment due to effort and relative success

### Data Analysis

The data used in the study included expressive language disorder scores of the subjects (in all three groups) in the pretest, posttest, and follow-up stages. Statistical methods, such as hypothesis testing and ANOVA, were used for data analysis. Data analysis was carried out through SPSS25.

### Ethical Considerations

The adequate level of confidentiality that has the right to withdraw from the study at any stage, the communication of research objectives with honesty and transparency and gaining the written consent of the subjects and their parents before the study, and use of the data only for the research were among the ethical considerations of the study. At the end of the study, the control group was also subjected to intensive intervention.

## Results

The subjects’ scores of expressive language disorder, including the mean and standard deviation, are presented for all three groups in the pretest, posttest, and follow-up stages. In the inferential statistics section, the ANOVA scores of all three groups in both posttest and follow-up stages were used to evaluate the intervention programs’ effectiveness and determine the difference between the groups.

According to Table 2, after the CBPT intervention, the mean and standard deviation values of expressive language scores of experimental group members in the posttest and follow-up stages have declined in comparison with expressive language disorders scores of students in the control and pseudo-control groups; in other words, the standard deviation of experimental group’s expressive language disorders was 16.350 and 6.722 in the pretest stage. However, these scores changed to 12.750 and 2.613 in the posttest stage and reached 12.200 and 2.667 in the follow-up stage, respectively. In the other two groups, the subjects only experienced a slight increase in the disorder’s severity.

**Table 2:** Descriptive indicators related to pretest, posttest, and follow-up stages of expressive language disorder tests.

Indicator	Experimental			Pseudo-control			Control		
	Follow-up	Posttest	Pretest	Follow-up	Posttest	Pretest	Follow-up	Posttest	Pretest
Number	20	20	20	20	20	20	20	20	20
Mean	12.200	12.750	16.350	13.750	12.700	12.050	13.100	12.650	12.100
SD	2.667	2.613	6.722	3.581	1.922	1.986	2.633	1.871	1.333

Before performing repeated-measures ANOVA, the homogeneity of variances measured by Box’s M test showed that the significance of Box’s M test (0.052) is higher than that of *α* = 0.001. Therefore, it can be argued that the assumption of homogeneity of covariance matrices is met. Kolmogorov–Smirnov test was used to test the normal distribution of data. The results show that the significance of the research variables exceeds 0.05; it can be argued that data are normally distributed in this variable.

Assessing the equality of variances using Levene’s test showed that the assumption of equality of variances is met and the error variance of dependent variable is equal in all groups [pretest = *F* (57.2) = 2.919, *p* = 0.064, posttest = F (57.2) = 0.426, *p* = 0.655, and follow-up = F (57.2) = 0.734, *p* = 0.484], which is not significant at 0.05. Therefore, it can be argued that the assumption of using ANOVA is met. In the data analysis section, repeated-measures ANOVA was used three times to measure expressive language disorder scores. The results showed that the significance of all aforementioned tests is below *α* = 0.05; therefore, repeated-measures ANOVA test can be used to test expressive language disorders in different groups. The Pillai’s Trace index of expressive language disorders was 0.031. Therefore, it can be argued that the effect of intervention time (pretest, posttest, follow-up), as well as the combined effect of time/group (control, pseudo-control, and experiment) on the mean expressive language disorders, is significant (Table 3). These results indicate the effectiveness of CBPT in improving students’ expressive language disorder. Therefore, repeated-measure ANOVA test results showed that the experimental group’s expressive language disorder has significantly changed in posttest and follow-up stages relative to the pretest stage.

**Table 3:** Results related to the effects between subjects on the variable of expressive language disorders.

Model	Power of test	Eta-squared	Sig	F-test	Mean square	Degree of freedom	Sum square
Time	1/000	0/982	0/000	3157/604	30758/939	1	30758/939
Time × group	0/948	0/175	0/039	3/300	22/406	2	44/811
Error	–	–	–	–	9/741	57	555/250

The Mauchly’s test of sphericity (*p* = 0.038) obtained a significance value of less than 0.05. Therefore, the null hypothesis (equality of the covariance matrix for dependent variables with the normalized distribution of an identity matrix) is rejected, and the sphericity hypothesis, which is one of the assumptions of repeated-measures ANOVA, is not met; therefore, the coefficient of Greenhouse–Geisser epsilon should be used. According to the value of this coefficient (0.901) that is very close to unity, it can be argued that Mauchly’s sphericity hypothesis is confirmed. In the next step, the repeated-measures ANOVA is used to check the significance of the difference between the scores of expressive language disorders, the assumption of normal data distribution, and the assumption of Mauchly’s sphericity as well as the equality of variances. The results of repeated-measures ANOVA are presented in Table 2.

The results of repeated-measures ANOVA are indicative of the significant effect of time and time × group [*F* (2, 57) = 3.30, *p* < 0.05]. Therefore, it can be argued that the difference addressed in the hypothesis is significant, and CBPT has had a significant impact on the expressive language disorders of children. The effect of intervention time (pretest, posttest, follow-up), as well as the effect of time ×group (control, pseudo-control, and experiment) on the mean expressive language disorder of children, is estimated to be significant (*p* < 0.05). Moreover, Eta-squared (effect size) indicates the effect of intervention time and group on the children’s expressive language disorders. Moreover, it can be argued that 98.2% of changes in the dependent variable (mean expressive language disorders) are imposed by the intervention time (pretest, posttest, follow-up), and the effect size for the time×group variable shows that 17.5% of the changes in the expressive language disorders are the result of these variables (time ×group). Powers of the test (1 and 0.94) are also indicative of significant accuracy of these causal relationships.

**Figure 1:**
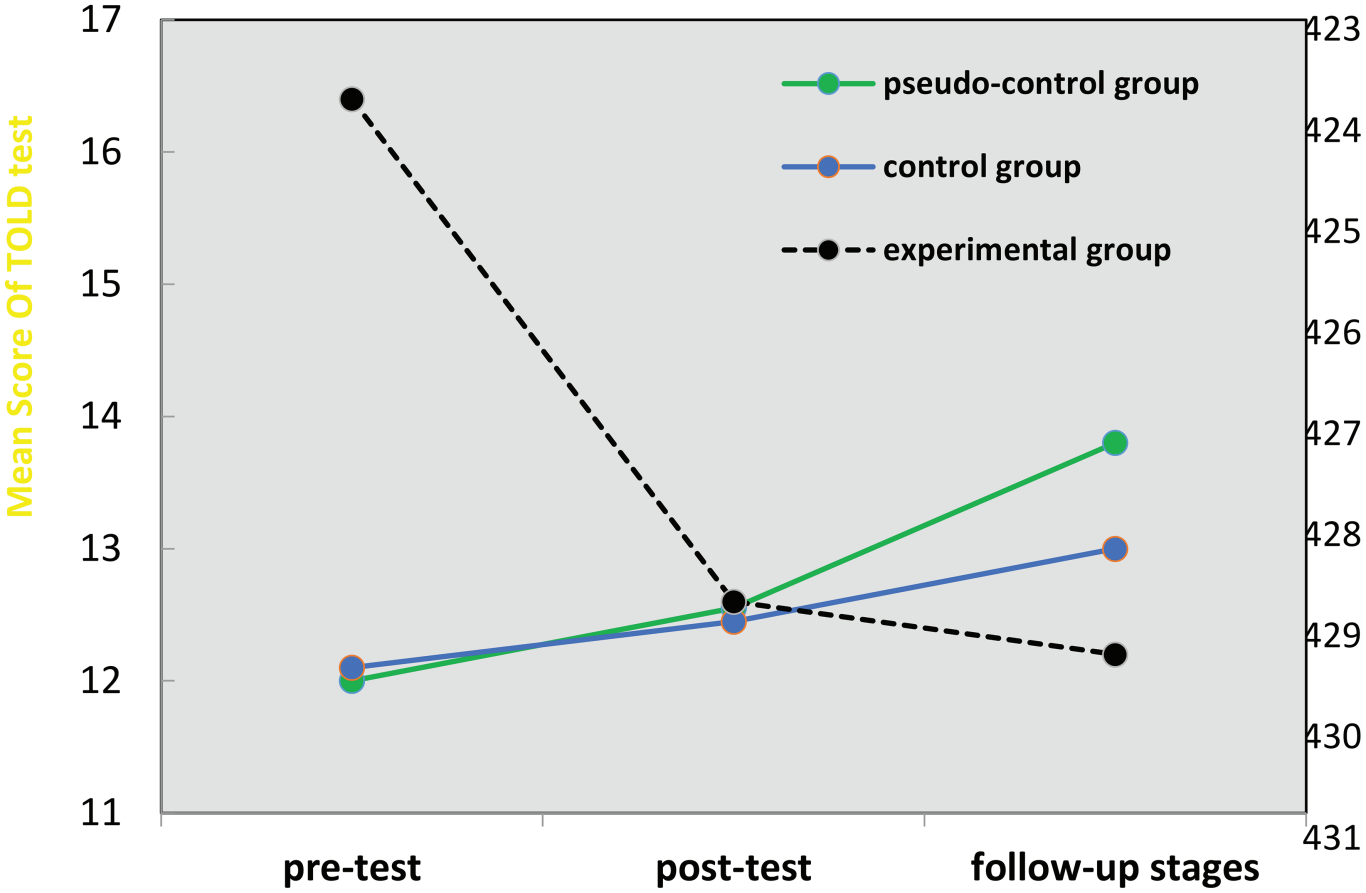
The difference among the mean expressive language disorders of students in all three groups during the pretest, posttest, and follow-up stages.

Taking into account the significant difference between the pretest, posttest, and follow-up scores in the experimental groups (*p* < 0.01), there is a pairwise comparison of the significant difference between the pre-intervention and post-intervention, and 2 months later there are intervention scores in experimental, control, and pseudo-control groups (mean difference, IJ), and the value of sig column in this Table 3 (0.005) is compared with *α* = 0.05. It can be argued that the control group is significantly different from the experimental group. Therefore, taking the intervention time into account, children’s expressive language disorders in the control group are expected to be much more intense than those in the experimental group. Therefore, the most significant difference between the control and the experimental groups is observable when the time variable is taken into account. Figure 1 also shows the difference between students’ mean expressive language disorder in the pretest, posttest, and follow-up stages. According to this figure, expressive language disorders of children in the control and pseudo-control groups have continuously intensified in the pretest, posttest, and follow-up stages, while expressive language disorders of students in the experimental group have continuously declined in the pretest, posttest, and follow-up stages.

## Discussion and Conclusion

In this study, we tried to explain how CBPT can improve expressive language disorders in bilingual children. The purpose of this study was to describe the experiences and understanding of cognitive–behavioral therapy in practice and provide an overview of cognitive–behavioral therapy interventions to create an effect on improving the expressive language disorders that were performed in bilingual children. Also, by investigations, researchers have been conducted until this research time on the effects of cognitive–behavioral therapy on the variable of improved language disorder in bilingual children did not achieve.

We examine the aspects of bilingual impact on children and components of cognitive–behavioral therapy interventions related to them, and we consider the limits of this study, while in this study, we consider practical consequences, theory, and future research. The results of this study showed that play therapy with a cognitive–behavioral approach affects the improvement of bilingual expressive language disorder. After presenting the treatment sessions with the cognitive–behavioral approach to bilingual children with expression language disorder, the group responded significantly more than two control and control groups to language test questions. What distinguishes the present research from similar internal and external researches is the use of a game-based cognitive–behavioral approach in an integrated format tailored to the focus on language.

### Bilingual Children

Children with language impairment, in addition to experiencing failures in learning, also experience unpleasant experiences, resulting from lack of proper communication and assertiveness in the community, which affects self-confidence, self-concept, and self-efficacy. Therefore, it is necessary to take action to solve the psychological and behavioral problems of these children. Therefore, the method introduced in this study according to the results obtained in Trevarthen and Aitken (2001) can be considered as a suitable method for using in general educational–rehabilitation programs of bilingual children, which will improve their expressive language skills (Vahab et al., 2012). Most learners complain that learning a second language is difficult and causes challenges and stress (Vahab et al., 2012), especially when they use it in various skills (speaking, writing, reading, listening) and in real-life situations (Akbari, 2015; Hwang et al., 2017), because this ability requires a lot of mental performance (Mehrani and Zabihi, 2017). A persistent debate in bilingual literature is whether learning two or more languages has cognitive benefits over the mere benefits of speaking a second language (Hassanzade and Minayi, 2002; Bialystok, 2017). Nichols et al. (2020) showed that evaluations among 11,000 participants in 12 executive functions whose functional and neurological characteristics were well described, bilinguals were superior to just one test of superiority (Nichols et al., 2020). Other studies also report no advantage of executive performance in bilingual (Lehtonen et al., 2018).

In our country, a large number of students with ethnic and native language proficiency and a little familiarity with the Persian language enter elementary school. That means that all of the children in the ages of 6 and 7 years the situation, the language, the same does not dominate them, to Persian, to a size of not, but from the past to the present educational system of our country, with all these children encounter linguistic actions have been. As there are many educational problems and lack of language proficiency in bilingual children hinders better learning, so bilingual students encounter different learning, emotional and educational problems.

### CBT Interventions for Improved Language Disorder

Cognitive-behavioral play therapy is a suitable and coherent program for children aged 3–8 years, and its main characteristic compared to other approaches is direct attention to objectives and planning specific methods for achieving educational goals, perception, and cognition, which is based on friendly, participatory, trust. Therefore, CBPT with the targeting of incompatible thoughts such as pulling photos, movie recording, and emotion cards increases compatibility beliefs.

The findings of the present study are consistent with several previous reports (Lewis et al., 2000; Riojas-Cortez and Flores, 2004; Danger and Landreth, 2005; Li, 2012). In other words, these studies confirm that play therapy improves language and expressive problems and develops expressive skills in children. Also, the results of the research show that a variety of plays are effective in L2 learning (Ang and Zaphiris, 2011; Reinhardt and Thorne, 2016; Li, 2021); for example, role-playing has many benefits in language learning (Efrizal, 2012), and it has been proven that role-playing with peers leads to language learning, speaking, and behavior modification (Masyitoh and Kaseh, 2018). Implementation of simulation plays also helps students in language learning to produce words, phrases, and sentences through language-based activities (Samah, 2013). Scientific evidence suggests that learning a second language through plays is more effective than learning without plays (Zarzycka-Piskorz, 2016). Therefore, it can be argued that the findings of the present study are consistent with the results of expressive language interventions carried out by other researchers and therapists. According to the present study findings, children who participated in CBPT programs are characterized by higher expressive language skills than children who did not participate in the program. In other words, the results showed that children experienced improvements in speaking or expressive language skills after receiving interventions. In line with our findings, previous researches also confirm the effectiveness of play therapy, especially CBPT (Leblanc and Ritchie, 2001; Lin and Bratton, 2015; Ray et al., 2015). During the treatment process, the therapist helps bilingual children with speech-language disorders to identify, modify, or construct their cognitions. By helping children identify their cognitive distortions, therapists teach children how to replace dysfunctional thoughts about school and the educational environment with functional thinking. In this study, children having problems with oral vocabulary definition, expression, word recall, and grammatical completion of sentences in the early intervention sessions, and even those who had problems introducing themselves to others or expressing their emotions and thoughts are managed, in the final sessions, to introduce themselves or their friend easily, narrate stories or events, and even express stressful situations and challenges. Among the children, some had problems in other areas such as linking words and semantics. They gained good scores in the final test, which indicated their success in overcoming the problems.

### The Strengths and Limitations of the Present Study

The findings of the present study show the importance of implementing cognitive–behavioral game interventions for bilingual children and show that these training include all aspects of language in which the child is weak, and after the training course these children can achieve the average level of language ability that this principle is necessary for their academic achievement and social communication. Also, the main strength of the study is the objective and their target population: Bilingual children often suffer a slight delay to reach linguistic milestones and therefore educators and parents may worry that they may present a disorder with an increase in such diagnosis among these children. Expressive language disorder is assessed in bilingual children as young as 6 years, and this timely intervention will make these children less likely to have problems in the future.

The present study has limitations since the present experimental study was not possible to control all interfering variables (parental literacy and their social and economic status and personality characteristics) and may be subjected to the underlying conditions that are out of control of the researcher’s control. And it can endanger the internal validity of the research, as well as the low partnership of parents and children in the intervention process and the follow-up period, it should be generalized in the generalization of caution and the generalization of the results of this research, subject to the views of population constraints, its cognition. The final limitation of studies in this study is the lack of longitudinal investigations.

### Recommendations for Future Research

In the end, researchers are advised to evaluate the effect of CBPT on other psychological variables in further studies. Speech and language therapists, consultants, and psychologists can use the designed play therapy protocol as a model and recognize it as a top-ranking treatment method. Cognitive–behavioral plays and activities can help bilingual children improve their linguistic skills and prepare them for joining others in society. Therefore, the inclusion of these plays in the intervention programs intended for bilingual children can contribute to their language disorders and complications. The present study can also be recognized as an introduction to assessing the effect of play therapy on language disorders in other groups of students including those suffering from learning disability, developmental delay, deafness, etc. and consequently contribute to the improvement of their mental and physical health. Therefore, as an independent course, play therapy should be included in the weekly curriculum of students, especially those living in the bilingual regions of the country, and CBPT should replace pointless plays.

## Conclusion

In the present intervention, group play therapy with a cognitive–behavioral approach uses more emotional, practical and nonverbal activities and theoretically emphasizes the interaction between the individual and the environment. Therefore, as expected, this program is effective in improving children’s language disorders. Another point is that the experience of different emotions in play therapy can be expressed in security and peace through imaginary symbols and toys. Play is also a way for a child to express their feelings, increase their relationships, share their experiences, reveal their dreams, and achieve self-fulfillment. Therefore, it is not unreasonable to expect that CBPT has a positive effect on expressive language disorders. Also, by participating in this type of play therapy, children can solve their problems and have the opportunity to express and expose their annoying feelings (Harris et al., 2019).

Focusing on the cognitive dimension, in CBPT, we tried to treat bilingual children with speech-language disorders. In this way, the child identifies distortions and learns to replace this maladaptive behavior with the school environment and educational environment. CBPT improved the level of expressive language skills of bilingual children, and this will pave the way for their success and development in the future.

## Data Availability Statement

The raw data supporting the conclusions of this article will be made available by the authors, without undue reservation.

## Ethics Statement

The studies involving human participants were reviewed and approved by Ferdowsi University of Mashhad. Written informed consent to participate in this study was provided by the participants’ legal guardian/next of kin.

## Author Contributions

All authors listed have made a substantial, direct and intellectual contribution to the work, and approved it for publication.

## Conflict of Interest

The authors declare that the research was conducted in the absence of any commercial or financial relationships that could be construed as a potential conflict of interest.

## Publisher’s Note

All claims expressed in this article are solely those of the authors and do not necessarily represent those of their affiliated organizations, or those of the publisher, the editors and the reviewers. Any product that may be evaluated in this article, or claim that may be made by its manufacturer, is not guaranteed or endorsed by the publisher.
